# A “Qualifyde” Practitioner of Floyd County on Boards, Colleges, Etc.

**Published:** 1896-02

**Authors:** 


### *EDITORIAL.*

---

The office of the Business Manager of THE JOURNAL is Nos. 311 and 312 Fitten building.

The editors are not responsible for views expressed by contributors.

Articles for publication should reach this office not later than the 15th instant.

Address all communications, and make all remittances payable to THE ATLANTA MEDICAL AND SURGICAL JOURNAL, Atlanta, Ga.

Reprints of articles will be furnished, when desired, at a nominal cost. Requests for the same should always be made *on the manuscript*.

---

### A "QUALIFYDE" PRACTITIONER OF FLOYD COUNTY ON BOARDS, COLLEGES, ETC.

We are in receipt of the following communication from a dark corner of Floyd county, in the region round about Kartah. We do not know exactly what pains this gentleman, but as he expresses a desire to see his "writeing" in print we take pleasure in gratifying him:

Editor Atlanta Medical and Surgical Journal Sir wee hav ben annoyd with your infurnal talk about an examining board for those hoo wish to go into the practic of medicen but wee dont hear you say any thang about examining those hoo are allredy in practic it is all rite if tha dont no any thang about their buisness Just so tha had M. D. to the last of their names. you may Start over the State of Georgia and half her grate faculty cannot tell you whear you will find the circul of willes nor whear you will find the pituitory boddeys and tha cant tell you the congugate diameter of the cavity of the normal femail pelvis. and you Mr Editor cant tell what purpos the appendic vermeformics Serves And an other thang you dont no is what purpos the thyroid glandes and superenial capsules serves I see in your Oct number of the Journal you hav given some questions and answers tho you failed to giv them from the list of the Georgia board that honorble board that a fiew of you soft headed tender footed literary nabobes imposed upon some of us poor Devles that had paid you the last sent of our money and you was not satisfyde with that. for you hav allreadey told two tales about what purpos your board would serve first it was to protect the people and next it was to protect the qualifyde practitioner and not either one of them was the trooth but Just to tell the trooth as you should hav done at the start it was to protect the medical colleges and fore a fiew of the colege pets to git some small feese and a little fame. for your honorble board dont cear any thang for the people nor for the honest doctor for your Georgia board let men pas and giv them licens to practice that dident know pots fracture from pots deseas of the spine and tha couldent describ collies fracture either and I hate to tell it but its the truith there was some one past that board that sed oun examination that tha would giv a child thirty drops of norways tincture of Veratrum but tha past the board OK. and you Still brag about your examining board now Mr. Editor I hav only rote the trooth and you cant deny what I hav Sed.

Yours in Medicen, nov the 29 * * *

Now Mr Editor wee would like to see this writeing spred over the pages of your famus Journal but that is somethang wee will never see as it is the trooth.

We regret very much to note that such profound knowledge of anatomy and physiology remains hidden with our esteemed and phonetic contributor in a remote precinct in Floyd county. What light he can throw upon the functions of the "appendic vermeformics," the "superenial" capsules, and the thyroid "glandes" should be given to an anxious profession at once, before his light goes out, so to speak. As for the examination of those "hoo are allredy in practic," we think that our sensitive friend, instead of agitating the matter, had better adopt the more prudent occupation of sawing wood; or, as the astute Brer Rabbit used to do, "lay low and say nothing."

We trust that the colleges and the boards will be able to continue their good work, in spite of the fierce comments of our esteemed correspondent.

